# NOX4 promotes non-small cell lung cancer cell proliferation and metastasis through positive feedback regulation of PI3K/Akt signaling

**DOI:** 10.18632/oncotarget.2025

**Published:** 2014-05-28

**Authors:** Cuixiang Zhang, Tian Lan, Jincai Hou, Juan Li, Rende Fang, Zhicheng Yang, Min Zhang, Jianxun Liu, Bing Liu

**Affiliations:** ^1^ Institute of Basic Medical Sciences of Xiyuan Hospital, China Academy of Chinese Medical Sciences, Beijing, China; ^2^ Beijing Key Laboratory of Pharmacology of Chinese Materia Medica, Beijing, China; ^3^ Vascular Biology Research Institute, Guangdong Pharmaceutical University, Guangzhou, China; ^4^ Department of Clinical pharmacy, School of Pharmacy, Guangdong Pharmaceutical University, Guangzhou, China; ^5^ Guangdong Key Laboratory of Pharmaceutical Bioactive Substances, Guangdong Pharmaceutical University, Guangzhou, Guangdong, China; ^6^ Department of health statistics, School of Public Health, Guangdong Pharmaceutical University, Guangzhou, China

**Keywords:** NOX4, non-small cell lung cancer, proliferation, metastasis, PI3K/Akt signaling

## Abstract

NADPH oxidase 4 (NOX4) is deregulated in various cancers and involved in cancer proliferation and metastasis. However, what the role of NOX4 plays during malignant progression of non-small cell lung cancer (NSCLC) remains unknown. Our results show that NOX4 was upregulated in NSCLC cell lines and samples from patients, compared with controls; NOX4 protein levels were closely correlated with clinical disease stage and survival time. Overexpression of NOX4 in A549 and H460 NSCLC cells enhanced cell proliferation and invasion *in vitro*, and produced larger tumors, shorter survival time, and more lung metastasis in nude mice than control cells. On the contrary, NOX4 depletion inhibited NSCLC cell aggressiveness. Inhibition of PI3K/Akt pathway could sufficiently block the cellular effects of NOX4 overexpression in NSCLC cells both *in vitro* and *in vivo*. Specifically, we demonstrated that PI3K/Akt pathway also positively regulated NOX4 expression via NF-κB-mediated manner. Therefore, there existed a mutual positive regulation between NOX4 and PI3K/Akt signaling in NSCLC cells, and NOX4 was confirmed to functionally interplay with PI3K/Akt signaling to promote NSCLC cell proliferation and invasion. In conclusion, the positive feedback loop between NOX4 and PI3K/Akt signaling contributes to NSCLC progression.

## INTRODUCTION

Lung cancer is the most common cause of cancer death worldwide [1]. Non-small cell lung cancer (NSCLC) accounts for up to 80% of all lung cancer cases, and patients usually present advanced disease at initial diagnosis [2]. Although recent advances in conventional therapies have yielded modest improvements in NSCLC patient outcomes, the 5-year survival rate remains at 15% [3]. Therefore, it is urgent to explore new and more effective targets against NSCLC malignant progression.

NADPH oxidases (nicotinamide adenine dinucleotide phosphate oxidase, NOXs) are a family of enzymes that have the primary function to generate superoxide or hydrogen peroxide. They consist of seven members, represented by different catalytic subunits: NADPH oxidase 1 (Nox1), Nox2 (gp91^phox^), Nox3, Nox4, Nox5, Duox1, and Duox2 [4]. NOXs have been confirmed to be correlated with progression of many diseases [5]. Especially for NOX4, the relative specific inhibitors such as GKT136901 and its derivatives have been developed by GenKyoTex with high safety profiles [6]. One derivative compound of GKT136901, named GKT137831, has been undergoing clinical evaluation for the treatment of diabetic nephropathy. Therefore, NOX4 may be a promising target in clinical use against many diseases.

Evidences in growing numbers have confirmed the close correlation of NOXs with cancer development and progression. Inhibition of some members of NOX families suppresses tumor growth and leads to cancer cell death [7,8]. In addition to regulation of cancer cell growth, NOXs also play important roles in cancer cell progression. For example, our previous study showed that inhibition of NOX activity significantly downregulates the expression of VEGF in NSCLC cell [9]. Liu et al. reported that ectopic expression of NOX1 enhances melanoma cell invasive ability through upregulation of MMP-2 expression and inducing epithelial-mesenchymal transition (EMT) [10]. Besides, inhibition of NOX activity was shown to suppress prostate cancer metastasis [11]. Especially for NOX4, it was the most frequently expressed NOX isoform in several tumor cell lines [4]. Many observations point to a critical role of NOX4 in apoptosis resistance and sustained tumor cell proliferation in various types of cancer cells including glioma cells [12] and pancreatic cancer cells [13]. Besides, NOX4 has been also shown to promote renal cell carcinoma cell invasion through hypoxia-induced interleukin 6-and 8-production [14]. In NSCLC cells, NOX4 has been also identified to be abundantly expressed [15]. However, what the role that NOX4 plays during NSCLC survival and progression remains to be elucidated.

In this study, to identify the potential role of NOX4 in NSCLC carcinogenesis, we first surveyed the expression of NOX4 in NSCLC patients and found a tight association of NOX4 expression with the poor clinical outcome of NSCLC patients. Such an association was further confirmed in NSCLC cell lines both *in vitro* and *in vivo*. Importantly, we find a positive feedback regulation between NOX4 and PI3K/Akt pathway, which promotes NSCLC cell aggressiveness.

## RESULTS

### NOX4 upregulation is associated with progression and poor prognosis of human NSCLC

**Figure 1:**
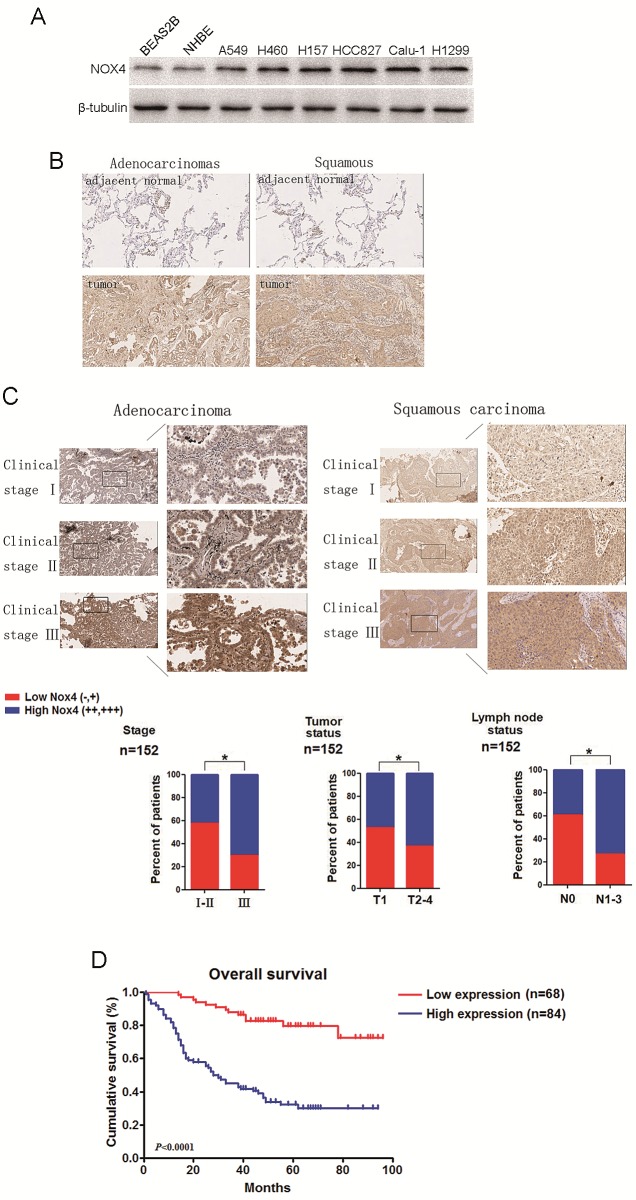
NOX4 is positively correlated with stage, tumor status, lymph node status, invasion and poor prognosis of NSCLCs (A) Western blotting analysis of NOX4 expression in normal lung epithelial cells and cultured NSCLC cell lines. (B-C) IHC staining indicating that NOX4 expression is upregulated in human NSCLCs (clinical stage I-III) compared with adjacent normal lung tissues. Percentage of patients with high expression of NOX4 and low expression of NOX4 according to different clinical parameters as follows: tumor stage, tumor status, and lymph node status. (D) Kaplan-Meier curves of NSCLC patiens with low vs high expression of NOX4 (n=152; *P*<0.001, log-rank test).

We first performed western blotting analyses to determine NOX4 expression phenotype in NSCLC cell lines and normal lung epithelial cells. The results revealed that NOX4 was markedly higher in NSCLC cell lines than that in normal lung epithelial cell lines (BEAS2B and NHBE cells). To determine the clinical significance of NOX4 expression in NSCLC patients, we analyzed samples from a cohort of 152 human patients with NSCLC using tissue microarray assay. The clinical patient information studied is summarized in Table 1. Immnohistochemical analysis showed that NOX4 was highly expressed in about 82% of NSCLC samples (125 of 152) both of adenocarcinoma and squamous carcinoma, whereas the adjacent normal tissues of NSCLC had much lower levels of NOX4 expression (Fig. 1B). The results of the IHC analysis are summarized in Table 2.

**Table 1:** Correlation Between the Clinicopathologic Features and Expression of NOX4

Patient characteristic	Numbers	NOX4 expression No (%)		*P* value
		(−,+)	(++,+++)	
Sex				
Male Female	82 70	38(46.3) 30(42.9)	44(53.7) 40(57.1)	0.776
Age (years)				
≤57 <57	73 79	35(48) 33(41.8)	38(52) 46(58.2)	0.477
Clinical stage				
I II III	35 44 73	25(71.4) 21(47.7) 22(30.1)	10(28.6) 23(52.3) 51(69.9)	0.0001
T classification				
T_1_ T_2_ T_3_ T_4_	76 29 37 10	40(52.6) 14(48.3) 12(32.4) 2(20)	36(47.4) 15(51.7) 25(67.6) 8(80)	0.0327
N classification				
N_0_ N_1_ N_2_ N_3_	79 24 43 6	48(72.1) 8(33.3) 11(25.6) 1(16.7)	31(27.9) 16(66.7) 32(74.4) 5(83.3)	>0.0001
Type				
Adenocarcinoma Squamous	82 70	36(43.9) 32(45.7)	46(56.1) 38(54.3)	0.887
Smoking history				
Smoking No smoking	76 76	31(41) 37(48.7)	45(59) 39(51.3)	0.32

**Table 2:** Overexpression of NOX4 in human NSCLCs

	Numbers	NOX4 expression No (%)		*P* value
		(−,+)	(++,+++)	
Ajacent non-tumor tissues	115	78(67.8)	37(32.2)	0.0016
NSCLCs	152	68(44.7)	84(55.3)	

Statistical analyses revealed that NOX4 expression was strongly correlated with the clinical stage and tumor-nodule-metastasis (TNM) classification in NSCLC patients both of adenocarcinoma and squamous carcinoma (Fig. 1C). Moreover, we found that patients with NSCLC with high NOX4 expression had shorter overall survival than those with low NOX4 expression (Fig. 1D). Taken together, these results indicate that NOX4 is positively correlated with poor prognosis in NSCLC patients and may be a potential predictive biomarker for disease outcome in NSCLC.

### NOX4 promotes the aggressiveness of NSCLC cells both *in vitro* and *in vivo*

**Figure 2:**
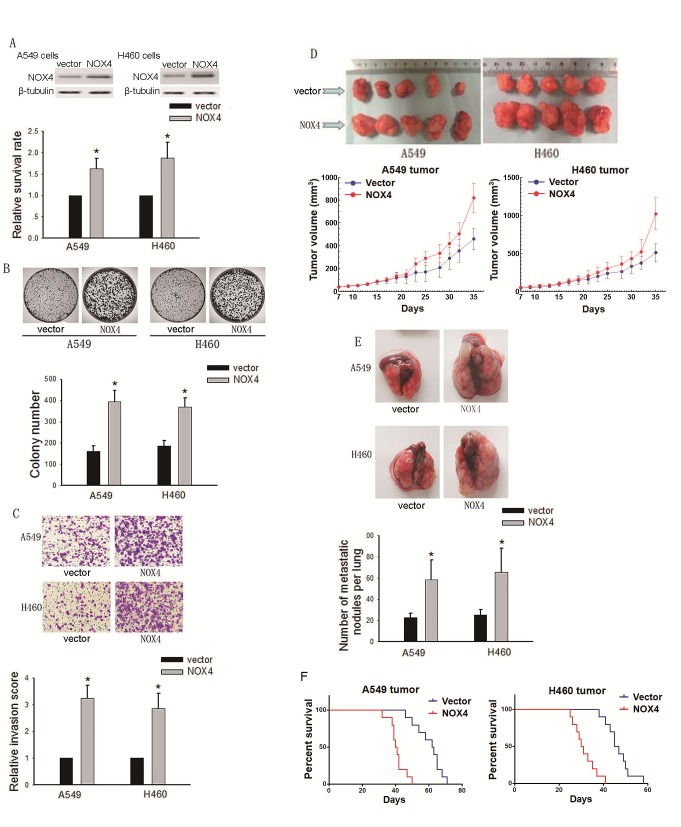
Effects of NOX4 overexpression on the aggressiveness of NSCLC cells both *in vitro* and *in vivo* (A-B) A549 and H460 cells were stably transfected with control vector, NOX4 plasmid, respectively. Overexpression of NOX4 in A549 and H460 cell lines analyzed by western blotting. β-tubulin was used as a loading control. NOX4 promotes the tumor growth of A549 and H460 cells evaluated using MTT assay (A) and colony formation assay (B). Bars are mean ± SD from four independent experiments. *Significantly different from vector control, *P*<0.05. (C) Cells were starved for 12 h before cell invasion assays were performed in presence of Matrigel transwell filters. The invaded cells were stained and counted. Bars are mean ± SD from four independent experiments. *Significantly different from vector control, *P*<0.05. (D) A549 and H460 cells stably expressing NOX4 or its control vector were transplanted into athymic mice (n=10 per group). Tumor size was measured every 2 days for indicated period. The representative tumors and growth curves of tumor are shown. (E) Representative images and number of metastatic nodules on the surface of the lungs of mice were injected with A549 and H460 stable transfectants (n=10 per group). Bars are mean ± SD. *Significantly different from vector control, *P*<0.05. (F) NOX4 regulates the survival rate of NSCLC tumor-bearing animals. Kaplan-Meier curves for illustration of the survival periods of A549 and H460 NOX4 overexpressing tumors-beared mice and their respective control group (n=10 per group).

To explore the effects of NOX4 on the proliferation and metastasis of NSCLC cells, A549 and H460 cells stably expressing ectopic NOX4 were established (Fig. 2A, upper panel). NOX4-transduced NSCLC cells displayed higher growth rates (Fig. 2A, lower panel) and increased anchorage-dependent growth (Fig. 2B) compared with vector-control cells. NOX4 overexpression also promoted the invasive ability of these cells as assessed by Matrigel invasion assays (Fig. 2C). The capability of NOX4 overexpression to promote NSCLC aggressiveness was also examined using an *in vivo* tumor model. As shown in Fig. 2D, both A549 and H460 tumors formed by NOX4-transduced NSCLC cells grew faster than vector-control tumors. After 12 weeks, mice injected with NOX4-transduced A549 and H460 cells displayed a statistically more number of lung metastasis than those injected with vector-control cells (Fig. 2E). Furthermore, NOX4 overexpression could significantly shorten the survival time of A549 and H460 tumor-harbored mice (Fig. 2F).

**Figure 3:**
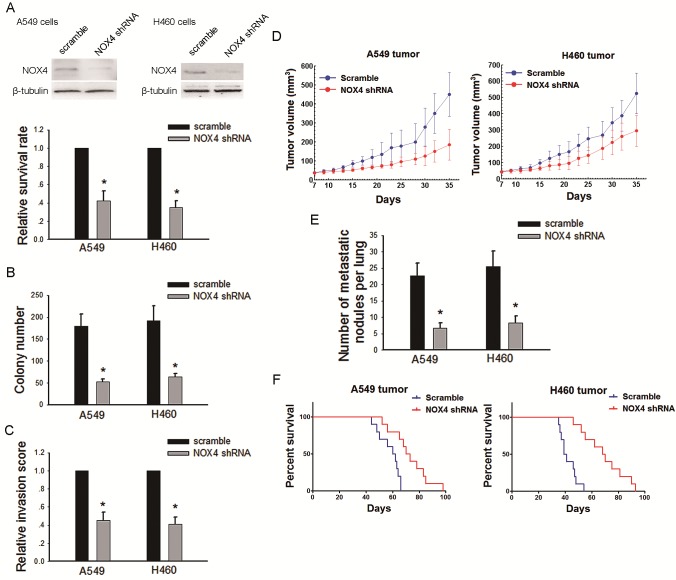
Silencing NOX4 inhibits the malignant properties of NSCLC cells both *in vitro* and *in vivo* (A-B) A549 and H460 cells were stably transfected with scramble shRNA, NOX4 shRNA, respectively. Knockdown of NOX4 in A549 and H460 cell lines was analyzed by western blotting. Knockdown of NOX4 inhibits the tumor growth of A549 and H460 cells evaluated using MTT assay (A) and colony formation assay (B). Bars are mean ± SD from four independent experiments. *Significantly different from vector control, *P*<0.05. (C) Cells were starved for 12 h before cell invasion assays were performed in presence of Matrigel transwell filters. The invaded cells were stained and counted. Bars are mean ± SD from four independent experiments. *Significantly different from vector control, *P*<0.05. (D) A549 and H460 cells stably transfecting NOX4 shRNA or its scramble shRNA were transplanted into athymic mice (n=10 per group). Tumor size was measured every 2 days for indicated period. The growth curves of tumor are shown. (E) Number of metastatic nodules on the surface of the lungs of mice were injected with A549 and H460 stable transfectants (n=10 per group). Bars are mean ± SD. *Significantly different from vector control, *P*<0.05. (F) NOX4 shRNA suppresses the survival rate of NSCLC tumor-bearing animals. Kaplan-Meier curves for illustration of the survival periods of A549 and H460 NOX4-depleting tumors-beared animal and their respective control group (n=10 per group).

To further confirm the role of NOX4 in the aggressiveness of NSCLC cells, we transfected NOX4 shRNA or control scrambles into A549 and H460 cells (Fig. 3A, upper panel). Depletion of NOX4 significantly decreased the growth (Fig. 3A, lower panel and Fig. 3B) and invasive capabilities (Fig. 3C) of NSCLC cells. The *in vivo* data showed that NOX4 shRNA-transfected A549 and H460 cells produced smaller tumors (Fig. 3D) and displayed much lower number of lung metastasis than control cells (Fig. 3E). Besides, NOX4 depletion could significantly prolong the survival time of tumor-harbored mice (Fig. 3F).

Collectively, these results indicate that NOX4 is necessary for the development of the aggressive phenotype of NSCLC cells.

### The involvement of PI3K/Akt pathway in NOX4-mediated aggressiveness of NSCLC cells

**Figure 4:**
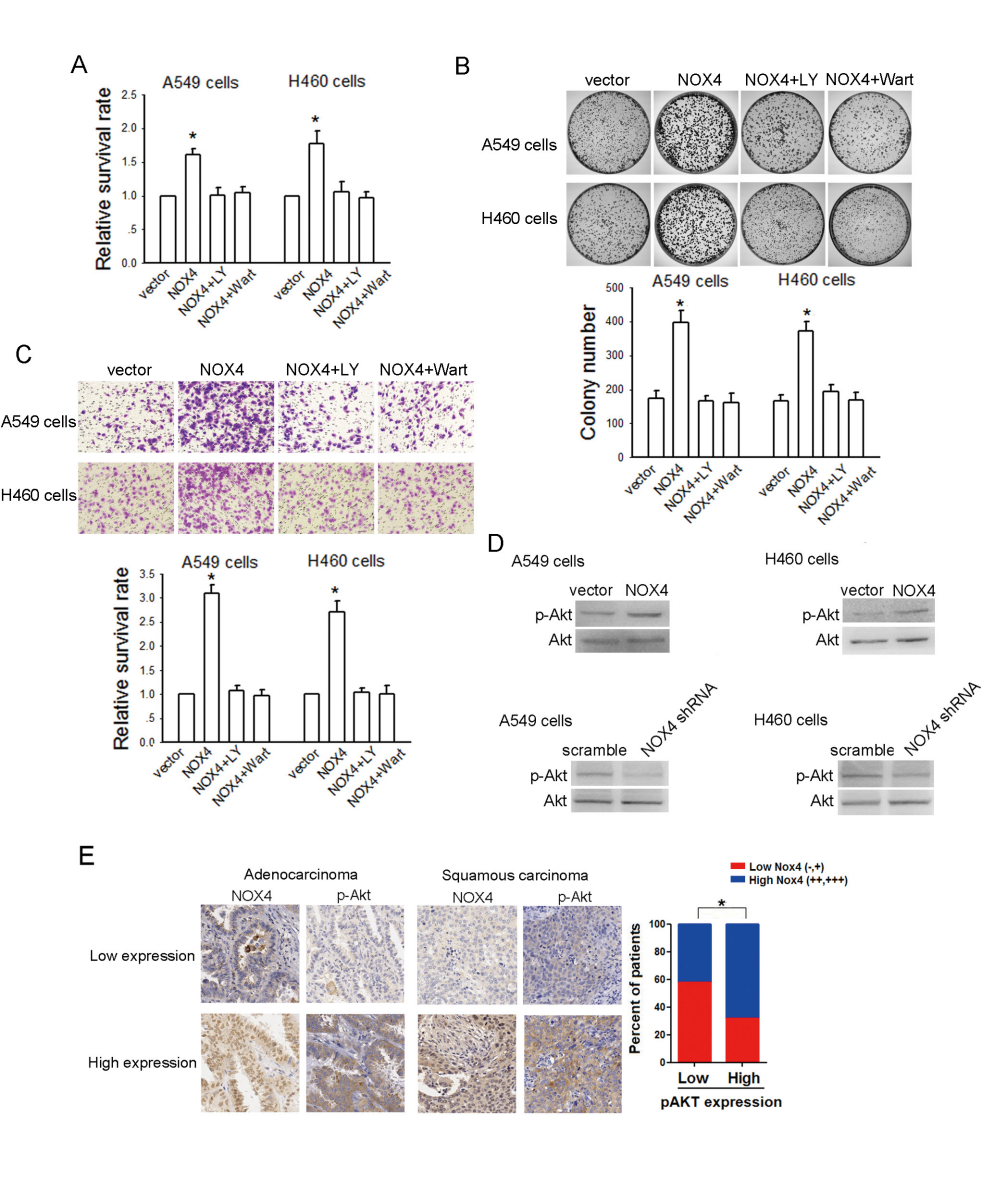
NOX4 promotes NSCLC progression through activating PI3K/Akt pathway *in vitro* (A-C) Stably NOX4 overexpressing A549 and H460 cells were treated with 30 μM of LY294002 or 10 μM of Wortmannin and control solvent. The proliferation of cells was evaluated using MTT assay (A) and colony formation assay (B). The invasion of cells was evaluated using Matrigel transwell assay (C). (D) NOX4 overexpressing or silencing led to increase or decrease in Akt activation in A549 and H460 cells measured by western blotting. Akt activity was represented as the levels of phosphorylated or total forms of Akt. Bars are mean ± SD from four independent experiments. *Significantly different from vector control, *P*<0.05. (D) NOX4 expression associated with pAkt expression in 152 primary human NSCLC specimens. Representative specimens with low and high levels of NOX4 are shown. * *P*<0.05.

PI3K/Akt pathway is the well documented downstream signaling of NOX [16,17]. Therefore, we sought to determine whether NOX4 -stimulated NSCLC aggressiveness is dependent on PI3K/Akt pathway. As shown in Fig. 4A,, treatment of A549 and H460 cells with a highly selective PI3K/Akt pathway inhibitor, LY294002 (30 μM), could block the enhancement effect of NOX4 on NSCLC cell growth and invasion. Comparable results were obtained from cells treated with another PI3K/Akt pathway inhibitor, wartmannin (10 μM). Furthermore, our results show that NOX4 significantly stimulated PI3K/Akt pathway as expressed by enhanced levels of phosphorylated Akt in A549 and H460 cells, while NOX4 depletion caused a reduction in PI3K/Akt activity (Fig. 4D). Moreover, the clinical correlation studies in 152 specimens showed that NOX4 levels were positively correlated with the expression of pAkt (Fig. 4E). Because NOX can also mediate many cellular events via activation of the MEK/Erk pathway especially in vascular endothelial cells [18], we performed experiments to explore whether MEK/Erk pathway is also involved in NOX4-promoted aggressiveness of NSCLC cells. The results showed that inhibition of MEK/Erk pathway by its specific inhibitor PD98509 (30 μM) had little or minimal effects on NOX4-mediated effects on NSCLC cells (data not shown). Taken together, these findings suggest that stimulation of PI3K/Akt pathway is sufficient to account for NOX4-promoted NSCLC cell aggressiveness *in vitro*.

**Figure 5:**
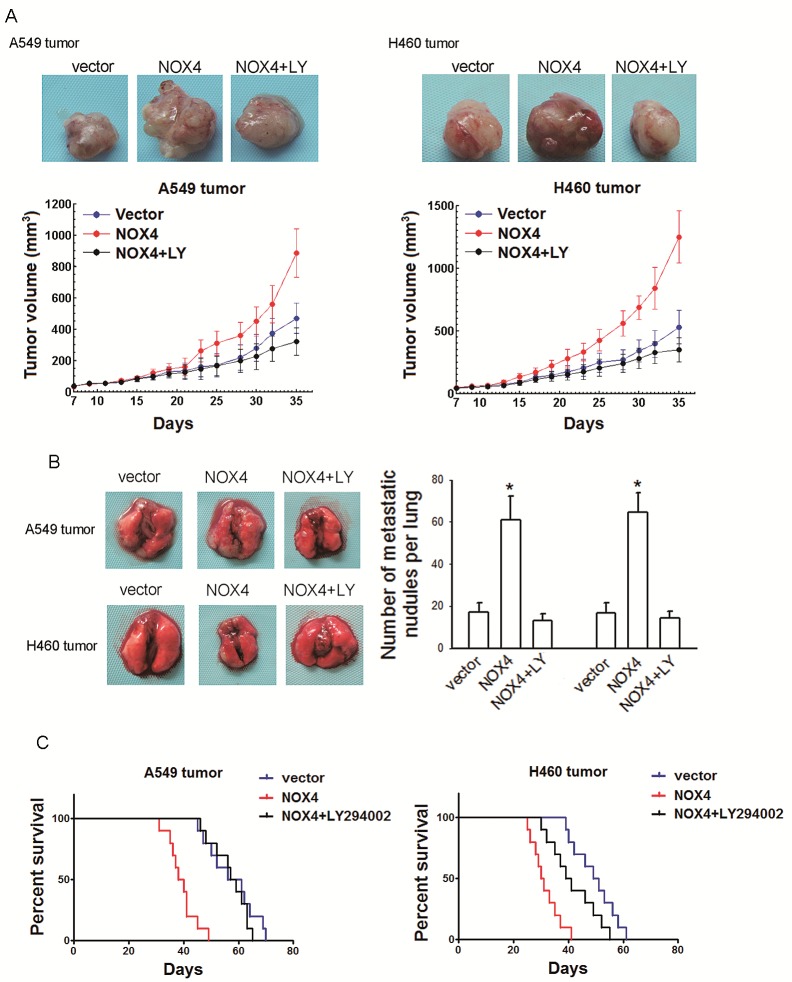
NOX4 promotes NSCLC progression through activating PI3K/Akt pathway *in vitro* (A-C) NOX4 overexpressing A549 and H460 tumors-bearing animals were treated with LY294002 (25 mg/kg, every four days, i.p.).The growth curves of tumor (A), number of metastatic nodules on the surface of the lungs of mice (B), and Kaplan-Meier curves for illustration of the survival periods (C) were respectively shown. Bars are mean ± SD. *Significantly different from vector control, *P*<0.05.

The *in vitro* data were confirmed by the *in vivo* results. Treatment with LY294002 (25 mg/kg, every four days, i. p.) reduced the tumor volume of NOX4-transduced tumor-harbored mice to the level comparable to that of vector-control group (Fig. 5A). Besides, inhibition of PI3K/Akt pathway could also reverse the effect of NOX4 on lung metastases (Fig. 5B) and survival time (Fig. 5C) *in vivo*.

### Reciprocal activation between NOX4 and PI3K/Akt pathway is critical for aggressiveness of NSCLC cells

**Figure 6:**
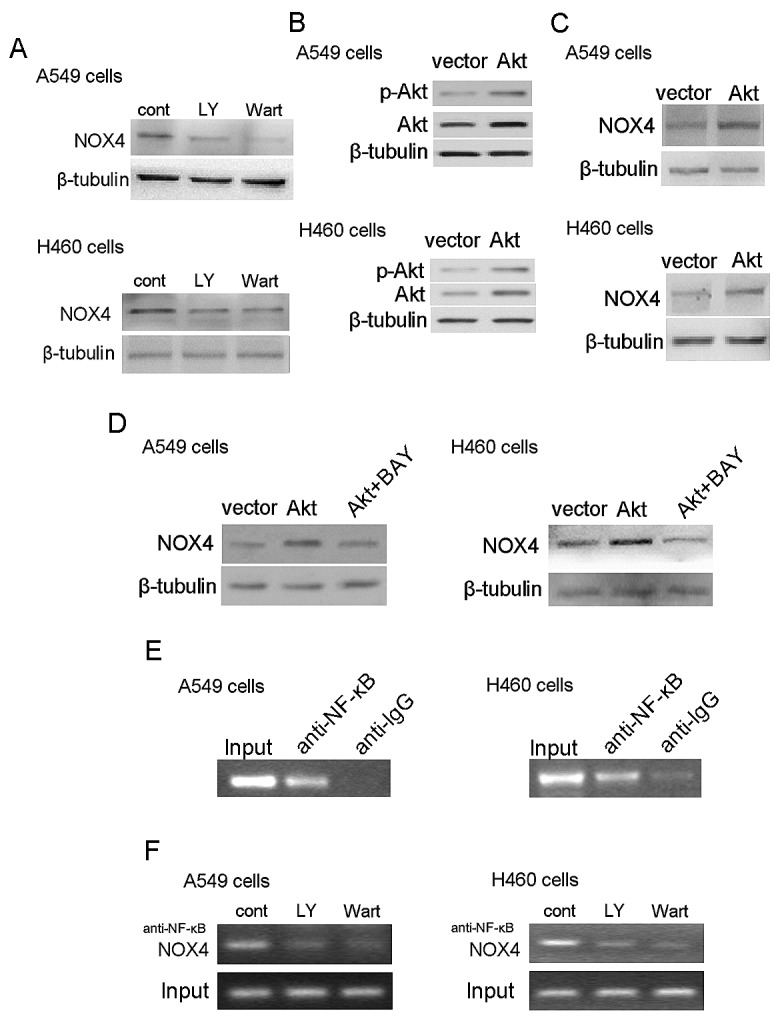
PI3K/Akt pathway regulates NOX4 expression in NSCLC cells through NF-κB NOX4 interplays with PI3K/Akt pathway to regulate NSCLC progression. (A) Western blotting analysis of NOX4 expression in A549 and H460 cells treated with 30 μM LY294002 or 10 μM Wortmannin and control solvent for 24 hour. (B) Overexpression of Akt in A549 and H460 cells. Akt activity was represented as the levels of phosphorylated forms of Akt evaluated by western blotting. (C) NOX4 expression was evaluated in Akt overexpressing A549 and H460 cells using western blotting. (D) Western blotting analysis of NOX4 expression in AKT-overexpressing A549 and H460 cells after treated with 10 μM NF-κB inhibitor (Bay11-7082). (E) The ChIP assay was performed using the chromatin prepared from A549 and H460 cells. IgG served as the negative control. (F) 30 μM LY294002 and 10 μM Wortmannin inhibited the direct binding of NF-κB to the promoter region of NOX4 and NOX4 expression measured by ChIP assay in A549 and H460 cells.

PI3K/Akt pathway is constitutively activated in many malignances including NSCLC [19,20]. To dissect whether Akt activation stimulates NOX4 expression as well, we first examined NOX4 expression after inhibition of PI3K/Akt pathway using LY294002 (30 μM) and wartmannin (10 μM) for 48 hours. As shown in Fig. 6A, treatment with LY294002 or wartmannin led to a significant decrease in NOX4 expression in A549 and H460 cells. To further confirm the role of PI3K/Akt pathway in regulation of NOX4 expression, A549 and H460 cells were transfected with pcDNA3.1-Akt plasmid. The transfection efficiency was confirmed by western blotting (Fig. 6B). Fig. 6C showed that Akt overexpression could substantially stimulate NOX4 expression in NSCLC cells. These data combined with that NOX4 can significantly stimulated PI3K/Akt pathway in NSCLC cells (Fig. 4D) demonstrate that there exists a reciprocal activation between NOX4 and PI3K/Akt pathway in NSCLC cells.

As NF-κB is the well-known downstream transcription factor, we sought to examine whether NF-κB is involved in Akt-stimulated NOX4 expression in NSCLC cells. As shown in Fig. 6D, BAY 11-7082 (10 μM) treatment efficiently blocked the enhancement effect of Akt overexpression on NOX4 expression in A549 and H460 cells determined by western blotting. Furthermore, the results of ChIP assays indicated that NF-κB directly bound the NOX4 promoter (Fig. 6E) and inhibition of PI3K/Akt signaling by LY294002 or wartmannin treatment significantly reduced the binding of NF-κB to the NOX4 promoter (Fig. 6F).

**Figure 7:**
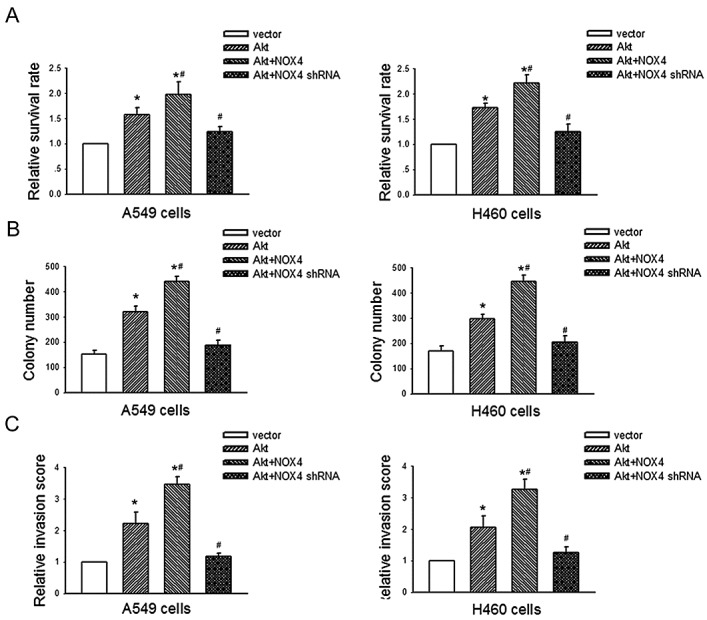
NOX4 interplays with PI3K/Akt pathway to regulate NSCLC cell proliferation and invasion A549 and H460 cells were transfected with Akt alone, or in the presence of NOX4 or NOX4 shRNA respectively. The proliferation of cells was evaluated using MTT assay (A) and colony formation assay (B). The invasion of cells was evaluated using Matrigel transwell assay (C). Bars are mean ± SD from four independent experiments. *Significantly different from vector control, *P*<0.05.

We further evaluated the biological significance of the reciprocal regulation between NOX4 and PI3K/Akt pathway in the growth and invasion of NSCLC cells. As shown in Fig. 7A,, Akt overexpression alone could also resulted in elevated growth and invasion of A549 and H460 cells. Moreover, NOX4 overexpression could further enhance Akt-promoted cell growth and invasion, whereas NOX4 knockdown rendered impaired Akt-mediated effects in these cells. Collectively, these results indicate that NOX4 and PI3K/Akt pathway are functionally interdependent in promoting NSCLC cell aggressiveness.

## DISCUSSION

The present study indicates that NOX4 is markedly upregulated in human NSCLC cells and tissues compared with normal cells and tissues. Statistical analysis of IHC staining reveals that NOX4 expression is positively correlated with clinical progression of NSCLC. NOX4 promotes NSCLC cell malignant progression both *in vitro* and *in vivo*, probably through activation of PI3K/Akt pathway. Specifically, we find that there is a reciprocal positive regulation between NOX4 and PI3K/Akt signaling in NSCLC cells, which functionally interplays to enhance aggressiveness of NSCLC cells.

The overall 5-year survival rate of NSCLC patients remains poor, which is largely due to the high rates of extensive local invasion and mediastinal nodal metastasis [21]. NSCLC patients develop mediastinal nodal metastasis at the early stage, and mediastinal nodal metastasis has been identified as the most important predictor of prognosis in NSCLC [22,23]. Herein, we found that NOX4 expression is strongly correlated with N and M classification, and NSCLC with high NOX4 expression has shorter overall survival than those with low NOX4 expression. These data suggest that NOX4 is associated with NSCLC metastasis and may be a potential predictive biomarker for disease outcome in NSCLC.

NOX4 has been confirmed to promote cell aggressiveness of various types of cancer. For example, NOX4 has been shown to protect pancreatic cancer cells from apoptosis [13] and mediate proliferation and survival of pancreatic cancer cells [24]. Besides, NOX4 is also involved in renal cell carcinoma cell invasion and metastasis through hypoxia-induced interleukin 6- and 8- production [14]. Nevertheless, what role of NOX4 in NSCLC cell aggressiveness and the underlying mechanisms remain unknown. In the present study, we found that overexpression of NOX4 increases NSCLC cell growth and invasion *in vitro*, as well as metastasis *in vivo*. Previous studies reported that inhibition of NOX4 is sufficient to activate apoptosis via PI3K/Akt pathway in pancreatic cancer PANC-1 cells [17], and NOX4 mediates many cellular effects in various types of normal cells [25,26]. Therefore, whether NOX4-stimulated NSCLC aggressiveness is dependent on PI3K/Akt pathway was determined. We found that inhibition of PI3K/Akt pathway reverses the effect of NOX4 on NSCLC growth and metastasis. Besides, NOX4 overexpression significantly stimulates PI3K/Akt pathway in NSCLC cells. MEK/Erk pathway is another important downstream signaling, however, we found that inhibition of MEK/Erk pathway has little or minimal effects on NOX4-mediated effects on NSCLC cells. These results indicate that NOX4 promotes NSCLC cell aggressiveness mainly through activation of PI3K/Akt pathway.

The mechanisms by which NOX4 stimulates PI3K/Akt pathway in NSCLC cells have not been explored. In many types of cells, NOX4-derived ROS can directly stimulate PI3K/Akt signaling [27,28]. NOX4-derived ROS can also inactivate PTEN [16,29], which is a well-known negative regulator of PI3K/Akt activity. Besides, NOX4 has been shown to promote the phosphorylation of EGFR [30,31], whose activation results in stimulation of PI3K/Akt pathway, and this effect may be, at least partly dependent on redox regulation of PTP1B [32]. These findings suggest that the mechanisms for NOX4-stimulated PI3K/Akt pathway are complicated and there may be crosstalk with other signals, like EGFR signaling, to further activate PI3K/Akt signaling.

Interestingly, in this study, we found that inhibition of PI3K/Akt pathway results in reduced NOX4 expression, and Akt overexpression can substantially increase NOX4 expression in NSCLC cells. These results combined with that NOX4 overexpression stimulates PI3K/Akt pathway demonstrate that there exists a positive feedback regulation between NOX4 expression and PI3K/Akt pathway in NSCLC cells. In-depth study, we found that inhibition of NF-κB by its specific inhibitor BAY 11-7082 sufficiently blocked the enhancement effect of Akt overexpression on NOX4 expression, and the results of ChIP assays showed that NF-κB directly bound the NOX4 promoter and inhibition of PI3K/Akt signaling significantly reduced the binding of NF-κB to the NOX4 promoter. These findings indicate that PI3K/Akt signaling positively regulates NOX4 expression via the NF-κB-mediated manner, which is not an ‘off-target’ effect.

PI3K/Akt pathway is constitutively activated in NSCLC [20]. Though amplification of the PIK3CA gene and loss-of-function mutations in PI3K/Akt pathway inhibitory genes, such as PTEN have been shown to account partly for constitutive activation of PI3K/Akt pathway [33], the comprehensive mechanisms remain largely unknown. In this study, our results that NOX4 is upregulated during NSCLC progression and NOX4 can stimulate PI3K/Akt pathway in a positive-feedback manner strongly suggest that NOX4 overexpression accounts for a novel mechanism of persistent PI3K/Akt activation in NSCLC cells.

Our study shows that NOX4 or Akt overexpression alone results in enhanced growth and invasion of NSCLC cells. Moreover, NOX4 overexpression further enhances Akt-promoted cell growth and invasion, whereas NOX4 knockdown renders impaired Akt-mediated effects in these cells. These results indicate that dysfunction of either Akt or NOX4 contributes NSCLC cell aggressiveness, and more importantly, the reciprocal activation between NOX4 and PI3K/Akt pathway may represent a positive regulatory loop that mutually reinforces the NOX4 expression and PI3K/Akt activity, further augmenting NSCLC cell aggressiveness.

Our work has some limitations. It remains to be investigated the comprehensive mechanisms by which NOX4 stimulates PI3K/Akt pathway in NSCLC cells. Furthermore, we did not confirm the functional interplay of NOX4 and PI3K/Akt pathway to promote NSCLC cell aggressiveness *in vivo* due to the very complex *in vivo* experimental systems. Notwithstanding these limitations, our study does demonstrate that NOX4 and PI3K/Akt pathway can reciprocally positively regulate each other, leading to enhanced NSCLC cell growth and invasion. Therefore, NOX4 may be a promising target against malignant progression of NSCLC.

## MATERIAL AND METHODS

### Materials

Wartmannin and LY294002 (PI3K inhibitors) and PD98059 (MEK inhibitor) were obtained from Merck. BAY 11-7082 (NF-κB inhibitor) was purchased from Sigma Aldrich (St. Louis, MO). Cell culture reagents were obtained from Invitrogen. All other reagents were from Sigmaunless stated otherwise.

### Retrospective analysis

Patients at the initial diagnosis of NSCLC at Xiyuan hospital (Beijing, China) between March 12, 2001 and October 15, 2004 were included in this study. Inclusion criteria were patients with primary NSCLC, having tumor stages I A to III A, having received surgery as initial treatment modality, and having complete clinicopathologic data. Clinicopathologic data included age, sex, smoking history, histopathologic diagnosis and pathologic tumor stages. Histologic diagnosis was assigned in accordance with the WHO criteria for lung and pleural tumors, and pathologic stage was according to the revised international system. Prior patient consent and approval from the Ethics Committee of Xiyuan hospital were obtained for the use of clinical specimens and information for research purposes.

### Specimen preparation and immunohistochemical analysis

The surgical NSCLC specimens and matched non-tumor adjacent tissues were fixed in buffered formalin (10% vol/formalin in water, PH 7.4) and embedded in paraffin wax. The archived specimens underwent immunohistochemical staining for analysis of protein expression. The primary NOX4 and p-Akt antibodies were applied to the slides and incubated at 4 °C overnight. The slides were washed and then stained with the secondary antibody and DAB disclosure. The degree of immunostaining of paraffin-embedded sections was scored independently by two observers, based on the intensity index of staining. The proportion of tumor cells was scored as follows:, 1 (< 10% postitve tumor cells), 2 (10%-50% positive tumor cells), and 3 (> 50% positive tumor cells). The intensity of staining was graded according to the following criteria: - (no staining); + (weak staining = light yellow), ++ (moderate staining = yellow brown), and +++ (strong staining = brown).

### Cell lines, plasmids, and transfection

Human NSCLC cell lines and normal lung epithelial cells (originally purchased from ATCC) were used. Cells were maintained at 37°C and 5% CO_2_ in Dulbecco';s modified Eagle';s medium (DMEM) supplemented with 10% fetal bovine serum (Gibco) and penicillin 100 (U/ml)/streptomycin (100 μg/ml). Stable cell lines expressing the NOX4 or shNOX4 were generated by transfection of pCMV-NOX4 or pRS-shNOX4 into A549 and H460 cells and screened for 10 days with 400 μg/ml G418 or 0.5 μg/ml puromycin 48h after transfection, respectively. For Akt plasmid transient transfection, A549 and H460 cell (60% confluence, approximately 5× 10^6^ cells) were tranfected with 2 μg of pcDNA3.1-Akt (Guangzhou Ribobio Co.) or pcDNA3.1 using lipofectamine 2000 (Invitrogen, Carlsbad, CA) according to the manufacturer';s instructions.

### Cell proliferation/viability assay

The protocols used for MTT assay (detection of cell proliferation/viability) were all according to our previous study with minor modifications [34]. 5×10^4^ cells in 100 μL of serum-free DMEM were seeded in 96-well and incubated for 48 hours. Then, MTT was added to each well (with a final concentration of 0.5 mg/ml). After incubation at 37°C for 4 h, the plates were centrifuged at 450 ×g for 5 mins. Untransformed MTT was removed by aspiration, and formazan crystals were dissolved in dimethyl suloxide (150 μl /well) quantified spectrophotometrically at 563 nm.

### Colony formation assay

Cells were plated in 6-well plates (5×10^2^ cells per plate) and cultured for 14 days. The colonies were stained with 1% crystal violet for 10 mins after fixation with 10% formaldehyde for 5 mins.

### Cell invasion assay

Cell invasion assay was performed according to our previous study [34]. The invasiveness of NSCLC cells was determined using Boyden chambers consisting of Transwell (Corning Costar Corp, Cambridge, MA) with 8 μm pore size polycarbonate membrane filters precoated with 50 μL of Matrigel (1.25 mg/mL). During MTT assay, equal NSCLC cells (5×10^4^ cells) of the second group suspended in the serum-free DMEM of 100 μL were seeded onto the upper chamber of Matrigel-coated filter inserts. Serum-containing DMEM (500 μL) was added to the lower chamber. After 48-hour incubation, filter inserts were removed from the wells and the cells on the upper surface of the filter were wiped with a cotton swab. Filters were fixed 4% paraformaldehyde for 30 mins and stained with 0.1% crystal violet for another 30 mins. Then cell invasion was determined as eight high-power fields of cells were counted in each well under an inverted microscope at 200× magnification. Cell invasion was presented as the relative invasive score of treated group (invaded cell number/total cell number assayed by MTT represented by OD570) divided by that of control group.

### Western blotting

Western blotting protocol was according to our previous reports [9,34]. Cells were first seeded at a concentration of 1×10^6^ cells per 100-mm dish (Corning), incubated at 37 °c for certain days according to each experiment. For Western blotting analysis of NOX4, anti-NOX4 rabbit polyclonal antibody (ab154244; Abcam, Cambridge, MA, USA) was used. For analysis of Akt and p-Akt, blots were probed with their specific antibodies (diluted with 5% BSA to 1: 1000; all antibodies from Cell Signaling). Membranes were probed with horseradish peroxidase (HRP)–labeled anti-rabbit secondary antibody (diluted with 5% BSA to 1: 1000; all antibodies from Cell Signaling). Antibody binding was detected by enhanced enhanced chemiluminescence detection kit (ECL) (UK Amersham International plc).

### Xenograft studies

Animal handling and procedures were approved by the Guangdong pharmaceutical university Institutional Animal Care and Use Committee. A549 and H460 cells (approximately 1x10^6^ cells) were subcutaneously inoculated into the right flank of 6-week-old female nude mice. Seven days later, LY294002 (25 mg/kg) was intraperitoneally injected into the nude mice every four days. Tumor sizes were calculated with the formula: (mm^3^) = (LxW^2^) x0.5. The tumor volume was measured every other day. The metastatic ability of A549 and H460 cells (2 × 10^6^ cells per cell line) was determined following cell injection intravenously into the tail vein (n=10 per group). After 12 weeks, the mice were sacrificed and the number of metastatic nodules on the lung surface was counted. Survival was recorded daily. The survival in a cycle was approximately 60 days.

### Chromatin Immunoprecipitation

ChIP assay was performed strictly according to the manufacturer';s instructions (Cell Signaling Technology). Briefly, 1× 10^7^ cells were treated with 1% formaldehyde for 15 mins, harvested, suspended in SDS-lysis buffer, sonicated. Following centrifugation, supernatants were collected, diluted, and precleared with salmon spermsaturated protein A (Zymed, San Francisco, CA) to remove nonspecific immunoglobulins. Immunoprecipitation was performed by adding 10 μg NF-κB antibody (Sigma, St. Louis, MO) or nonimmune IgG (negative control) per reaction. Complexes were washed with low and high salt buffers, and the DNA was extracted and precipitated. The primers used for putative NF-κB binding element on the NOX4 promoter were forward 5';-gcttt agttt gggag tggga-3'; and reverse 5';-gaaat ttgag ccggaaacag-3';. Nonimmunoprecipitated chromatin fragments were used as an input control.

### Statistical analysis

Statistical analysis was evaluated by Student';s test for simple comparisons between two groups and one-way ANOVA for comparisons among multiple groups using JMP7.0 software (SAS Institute Inc, Cary, US). The relationship between NOX4 expression and clinicopathologic characteristic was analyzed using the chi-square test. Survival curves were plotted by the Kaplan-Meier method and compared using the log-rank test. Survival data were evaluated by using univariate and multivariate Cox regression analyses. All data are expressed as mean ± S.D. *P* value of < 0.05 was considered statistically significant.
